# Books and Magazines

**Published:** 1903-11

**Authors:** 


Something for Leisure Hours.—"Stories of a Country Doctor," and "Recollections of a Rebel Surgeon," are two books that every doctor will want to add to his library. The wit, pathos, anecdote and personal experience will appeal to the heart of every brother in the profession. The tales are told with a spontaneous, brilliant style of expression, and the interest is quickened and held from first to last. Don't fail to take advantage of our special offer: The two books, bound in cloth, for only $1.00. The above books shipped upon receipt of price. Your money back if not satisfied. The Clinic Publishing Company, Ravenswood Station, Chicago.

A Text-Book of Diseases of the Eye.—A Hand-book of Ophthalmic Practice for Students and Practitioners. By G. E. De Schweinitz, A. M., M. D., Professor of Ophthalmology in the University of Pennsylvania, etc. Fourth edition, revised, enlarged, and entirely reset. Octavo volume of 773 pages, with 280 illustrations and six chromo-lithographic plates. Cloth, $5.00 net; sheep or half Morocco, $6.00 net.

This book has attained its fourth edition, which is sufficient proof of its deserved popularity. Written in the hope that it would prove of service to both students and practitioners, it has more than fulfilled all expectations. The methods of examining the eyes, and the symptoms, diagnosis, and treatment of ocular diseases have received the largest share of attention. The subject matter has been given in greater detail than is customary in books of its scope, doubtless because the author, being a teacher of wide experience, recognized more fully than others, the knowledge requisite for the successful practice of ophthalmic science. In this new edition the text has been thoroughly revised, and the entire work has been reset, many new chapters have been added, such as Thomson's Lantern Test for Color-Blindness; Hysteric Alopecia of the Eyelids; Metastatic Gonorrheal Conjunctivitis; Grill-like Keratitis (Haab); the so-called Holes in the Macula; Divergence-paralysis; Convergence-paralysis, and many others. A large number of therapeutic agents comparatively recently introduced, particularly the new silver salts, are given in connection with the diseases in which they are indicated. The illustrative feature of the work has been greatly enhanced in value by the addition of many new cuts and six full-page chromo-lithographic plates, all most accurately portraying the pathologic conditions which they represent. There is no question that this fourth edition will attain the same popularity as did its predecessors.—*Ex.*

